# Heat Exposure and Dementia-Related Mortality in China

**DOI:** 10.1001/jamanetworkopen.2024.19250

**Published:** 2024-06-28

**Authors:** Ya Gao, Lin Lin, Peng Yin, Haidong Kan, Renjie Chen, Maigeng Zhou

**Affiliations:** 1 School of Public Health, Key Lab of Public Health Safety of the Ministry of Education, NHC Key Lab of Health Technology Assessment, Fudan University, Shanghai, China; 2 National Center for Chronic Noncommunicable Disease Control and Prevention, Chinese Center for Disease Control and Prevention, Beijing, China; 3 School of Public Health, Hengyang Medical School, University of South China, Hengyang, Hunan, China

## Abstract

**Importance:**

Although existing research has found daily heat to be associated with dementia-related outcomes, there is still a gap in understanding the differing associations of nighttime and daytime heat with dementia-related deaths.

**Objectives:**

To quantitatively assess the risk and burden of dementia-related deaths associated with short-term nighttime and daytime heat exposure and identify potential effect modifications.

**Design, Setting, and Participants:**

This case-crossover study analyzed individual death records for dementia across all mainland China counties from January 1, 2013, to December 31, 2019, using a time-stratified case-crossover approach. Statistical analysis was conducted from January 1, 2013, to December 31, 2019.

**Exposures:**

Two novel heat metrics: hot night excess (HNE) and hot day excess (HDE), representing nighttime and daytime heat intensity, respectively.

**Main Outcomes and Measures:**

Main outcomes were the relative risk and burden of dementia-related deaths associated with HNE and HDE under different definitions. Analysis was conducted with conditional logistic regression integrated with the distributed lag nonlinear model.

**Results:**

The study involved 132 573 dementia-related deaths (mean [SD] age, 82.5 [22.5] years; 73 086 women [55.1%]). For a 95% threshold, the median hot night threshold was 24.5 °C (IQR, 20.1 °C-26.2 °C) with an HNE of 3.7 °C (IQR, 3.1 °C-4.3 °C), and the median hot day threshold was 33.3 °C (IQR, 29.9 °C-34.7 °C) with an HDE of 0.6 °C (IQR, 0.5 °C-0.8 °C). Both nighttime and daytime heat were associated with increased risk of dementia-related deaths. Hot nights’ associations with risk of dementia-related deaths persisted for 6 days, while hot days’ associations with risk of dementia-related deaths extended over 10 days. Extreme HDE had a higher relative risk of dementia-related deaths, with a greater burden associated with extreme HNE at more stringent thresholds. At a 97.5% threshold, the odds ratio for dementia-related deaths was 1.38 (95% CI, 1.22-1.55) for extreme HNE and 1.46 (95% CI, 1.27-1.68) for extreme HDE, with an attributable fraction of 1.45% (95% empirical confidence interval [95% eCI], 1.43%-1.47%) for extreme HNE and 1.10% (95% eCI, 1.08%-1.11%) for extreme HDE. Subgroup analyses suggested heightened susceptibility among females, individuals older than 75 years of age, and those with lower educational levels. Regional disparities were observed, with individuals in the south exhibiting greater sensitivity to nighttime heat and those in the north to daytime heat.

**Conclusions and Relevance:**

Results of this nationwide case-crossover study suggest that both nighttime and daytime heat are associated with increased risk of dementia-related deaths, with a greater burden associated with nighttime heat. These findings underscore the necessity of time-specific interventions to mitigate extreme heat risk.

## Key Points

**Question:**

Do nighttime and daytime heat exposure differ in their association with dementia-related deaths?

**Findings:**

In this case-crossover study of 132 573 dementia-related deaths, immediate and significant risk was associated with both nighttime and daytime heat. Stricter thresholds for nighttime heat were associated with a higher burden of dementia-related deaths compared with daytime heat.

**Meaning:**

These findings suggest that tailored public health interventions at different times of the day are critical for aging populations to effectively mitigate risk associated with extreme heat.

## Teaser

This case-crossover study assesses the risk of dementia-related deaths associated with short-term nighttime and daytime heat exposure.

## Introduction

Dementia, characterized by cognitive impairment and functional decline, has become a significant global public health concern.^1^ The reported global prevalence of dementia reached 43.8 million in 2016, an increase from 20.3 million cases reported in 1990.^2^ This number is projected to increase to an estimated 152 million individuals by 2050.^3^ The aging population contributes to this increasing prevalence, placing a burden on health care systems and society at large.^4^ Concurrently, climate change emerges as an urgent global challenge with profound effects on human well-being and health.^5^ Extensive research has documented the adverse effects of extreme heat on various health outcomes, including respiratory diseases, cardiovascular events, and mental disorders.^6,7,8,9^

The association between ambient temperature and dementia-related outcomes has garnered research attention. Prior studies have suggested that extreme high temperatures may be associated with elevated risk of cognitive decline and dementia-related morbidity and mortality.^10,11,12,13,14^ Various potential mechanisms have been proposed, including oxidative stress, excitotoxic effects, neuroinflammation, apoptosis, and alterations in blood flow and brain metabolism.^15,16,17,18^ However, existing findings are heterogenous and subject to some limitations. For example, the studied population is generally restricted to developed countries.^19,20^ In addition, most of the evidence was derived from ecological time-series studies that used daily aggregate hospitalization or mortality data, leading to potential ecological fallacy and residual bias from individual-level confounders.^21^

Recent attention has focused on the consequences of nighttime heat exposure, revealing significant associations between nighttime heat exposure and mortality, accelerated by climate change.^22,23,24,25^ Traditionally, nighttime temperatures provided relief from daytime heat, aiding individuals in recovering and adapting to elevated temperatures. However, the increasing trend in elevated nighttime temperatures raises concerns as it may disrupt sleep patterns, increase heat stress, and pose potential harm to vulnerable populations, especially older adults with dementia.^23,26^ Climate change projections warned of increasing temperature extremes, highlighting the urgency in identifying specific risk associated with different times of the day. However, there is a research gap concerning the potentially differential associations of nighttime and daytime heat with dementia-related deaths. This knowledge gap is of public health and clinical relevance given the distinctive attributes of nighttime and daytime temperatures, including factors such as intensity, duration, and their specific influences on human physiology.

In this nationwide study, we conducted an individual-level case-crossover study to investigate the association of nighttime and daytime heat exposure with dementia-related deaths. We aimed to quantitatively assess the risk and burden of dementia-related deaths and to identify potentially sensitive populations and regions.

## Methods

### Study Population and Mortality Data

We obtained mortality data from the China Cause of Death Reporting System (CDRS), a government-established system renowned for its adherence to stringent protocols, standardized procedures, and rigorous quality control measures. The CDRS is widely adopted by the central government for generating official mortality statistics to guide health policy, serving as a reliable source for scientific research.^27^ We extracted records of individual dementia-related deaths (*International Statistical Classification of Diseases and Related Health Problems, Tenth Revision* [*ICD-10*] codes F00-F03, G30, and G31) for all 2844 county-level administrative districts in mainland China spanning from January 1, 2013, to December 31, 2019, based on the underlying cause of death. In addition, demographic information, including sex, age, educational level, date of death, and residential addresses, was collected. The institutional review board of the School of Public Health at Fudan University exempted this study from review and waived the requirement for informed consent due to its analysis of deidentified data. This study followed the Strengthening the Reporting of Observational Studies in Epidemiology (STROBE) reporting guideline.

### Exposure Data

Hourly air temperature data were sourced from the fifth-generation atmospheric reanalysis product (ERA5), developed by the European Center for Medium-Range Weather Forecasts. ERA5 offers high spatial and temporal resolution, with the ERA5-Land dataset using data assimilation techniques to produce temperature series closely aligned with measurements from weather stations.^28^ We extracted hourly temperature series for all counties from the nearest ERA5-Land grid cell. Subsequently, we quantified residential exposures to daily mean temperature, nighttime heat, and daytime heat for each grid cell. The definitions of *daytime* and *nighttime* are provided in eMethods 1 in Supplement 1. In addition, daily air pollutant concentrations were collected from the National Urban Air Quality Real-Time Publishing Platform of China to allow for potential adjustments for air pollution effects. Our study focused on the warmest 5 months, spanning from May to September, to specifically investigate heat-related death risk during this period.

### Calculation of Hot Night Excess

To assess the intensity of nighttime thermal stresses, we used the hot night excess (HNE) metric, computed as the sum of nighttime thermal excesses using Equation 1^22,25^:
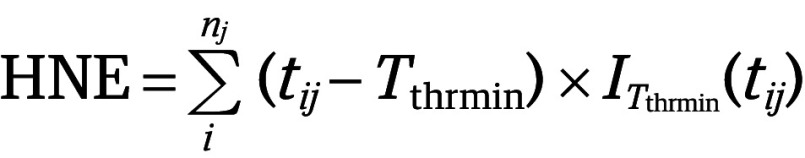
where *n_j_* represents the total nighttime hours on day *j*, *t_ij_* corresponds to the nighttime temperature at hour *i* on day *j*, *T*_thrmin_ indicates the local minimum temperature threshold beyond which the night is considered hot, and *I*_thrmin_ is determined as follows:



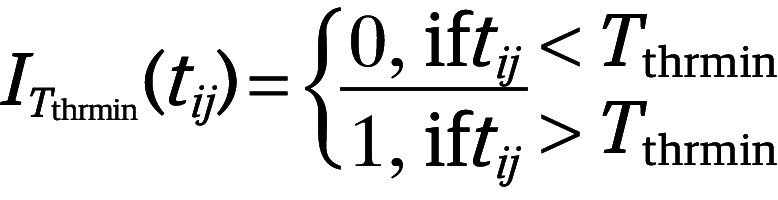



To establish the thresholds for identifying hot nights, we referred to daily minimum temperatures, which represent the coolest part of the day, typically occurring at night. Specifically, we considered different thresholds, such as the 90th, 92.5th, 95th, and 97.5th percentiles of the daily minimum temperature during the historical period (May to September, 2013-2019).

### Calculation of Hot Day Excess

Similarly, we computed the hot day excess (HDE) as a measure of daytime thermal stress intensity using the formula:
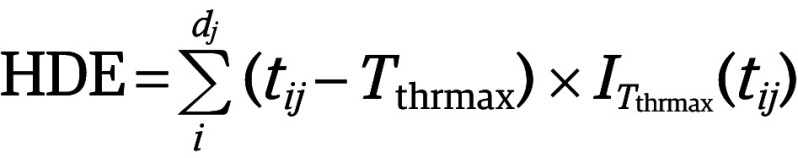
where *d_j_* represents the total daytime hours of day *j*, *t_ij_* corresponds to the daytime temperature at hour *i* on day *j*, *T_thrmax_* signifies the local maximum temperature threshold beyond which the day is considered hot, and *I*_thrmax_ is calculated as follows:



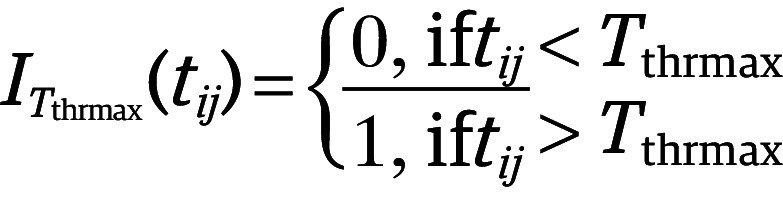



To determine the thresholds for identifying hot days, we relied on daily maximum temperatures, which provide a measure of peak heat stress experienced during the day. We considered various percentiles (eg, 90th, 92.5th, 95th, and 97.5th) from the historical period’s data.

### Statistical Analysis

Statistical analysis was conducted from January 1, 2013, to December 31, 2019. We used an individual-level, time-stratified case-crossover design to investigate the association between nighttime and daytime heat exposure and dementia-related deaths. Detailed descriptions of the design are provided in eMethods 2 in Supplement 1. Conditional logistic regression modeling integrated with distributed lag nonlinear modeling (DLNM) were used to analyze the data, considering various definitions of extreme heat. To establish exposure-response associations, we integrated cross-basis functions of HNE and HDE provided by DLNM into the conditional logistic regression model.^29^ Model selection was guided by the Akaike information criterion to ensure optimal parameter fitting. The cross-basis functions each featured a quadratic B-spline function with 2 interior knots placed at the 50th and 90th percentiles of the national HNE and HDE distributions, respectively, which could capture the potential nonlinear exposure-response associations.^22^ Lag-response associations were modeled using 3 inner knots at equal intervals on a logarithmic scale, with a maximum lag of 14 days.^30^ In addition, the model encompassed a cross-basis function for daily mean temperature with the same parameter settings to control for nonlinear and lagged confounding effects of daily mean temperature. Furthermore, the model included a categorical variable for holidays and a natural cubic B-spline for relative humidity with 3 degrees of freedom. To mitigate statistical uncertainty arising from small sample sizes and wider confidence intervals for extreme exposures, we restricted the display of these associations to the 1st percentile to the 99th percentile of the temperature series. The results are presented as the odds ratios (ORs) of dementia-related deaths and the corresponding 95% CIs for various definitions of extreme HNE and HDE compared with reference values, which correspond to the value with the lowest risk.

To assess the burden of dementia-related deaths associated with various definitions of HNE and HDE, we adopted a backward-looking approach as previously proposed.^31^ This approach offers precise estimations of the disease burden by accounting for complex lag patterns in temperature-related risk. We computed the attributable fraction (AF) at the national level and determined the empirical confidence intervals (eCIs) of AF using Monte Carlo simulations.^31^ Further details on the calculation procedures are available in eMethods 3 in Supplement 1.

We conducted a supplementary analysis to examine various subtypes of dementia according to specific *ICD-10* codes: Alzheimer disease (codes F00 and G30), vascular dementia (code F01), and unspecified dementia (codes F02, F03, and G31). To explore potential effect modifications, we conducted stratified analyses based on age (≤64, 65-74, and ≥75 years), sex (male vs female), educational level (middle school or less vs high school or more), and region (south vs north).

In addition, we conducted 2 sensitivity analyses. First, we adjusted for the 3-day mean of fine particulate matter and ozone to assess potential confounding by concomitant exposures to main air pollutants. Second, we included mutual adjustments for HNE and HDE in the main models.

All statistical analyses were conducted using R software, version 3.6.1 (R Project for Statistical Computing). Local sunset and sunrise hours on different days were derived from the suncalc package, version 0.5.1 in R. Statistical analysis was performed using the dlnm package, version 2.4.7, and survival package, version 3.2.7 in R. All tests were 2-sided and results were deemed statistically significant at *P* < .05.

## Results

### Descriptive Data

As depicted in eTable 1 in Supplement 1, our evaluation encompassed a total of 132 573 dementia-related deaths (mean [SD] age, 82.5 [22.5] years; 73 086 women [55.1%] and 59 487 men [44.9%]). Most deaths (108 142 [81.6%]) occurred in individuals aged 75 years or older. In addition, a substantial proportion of individuals (83 632 [63.1%]) lived in southern China. Among the dementia-related deaths, Alzheimer disease accounted for 24.0% (n = 31 804), vascular dementia accounted for 12.7% (n = 16 873), and unspecified dementia accounted for 63.3% (n = 83 896). The median for mean temperatures at all locations of deaths during the study period was 24.5 °C (IQR, 20.4 °C-25.7 °C). In addition, the median for daily maximum temperatures was 28.7 °C (IQR, 25.2 °C-30.0 °C), while the median for daily minimum temperatures was 20.3 °C (IQR, 15.5 °C-22.3 °C). Correlation analysis revealed a weak association between HNE and HDE with daily mean temperature. Specifically, the coefficient of determination (*R*^2^) was 0.26 for HNE and 0.19 for HDE when using a 95% threshold.

The eFigure in Supplement 1 illustrates the variations in hot night and hot day conditions, including mean thresholds and mean HNE and HDE, under different definitions for all counties in mainland China during the study period. The thresholds gradually increased, while the HNE and HDE decreased in all regions as the definitions became more stringent. Furthermore, the thresholds were higher in the southern regions, while the mean HNE and HDE were higher in the northern regions. For instance, when the threshold is defined as 95%, the median for the hot night threshold was 24.5 °C (IQR, 20.1 °C-26.2 °C), the HNE was 3.7 °C (IQR, 3.1 °C-4.3 °C), the hot day threshold was 33.3 °C (IQR, 29.9 °C-34.7 °C), and the HDE was 0.6 °C (IQR, 0.5 °C-0.8 °C) (eTable 2 and eTable 3 in Supplement 1).

### Results of Regression Analyses

**Figure 1.**
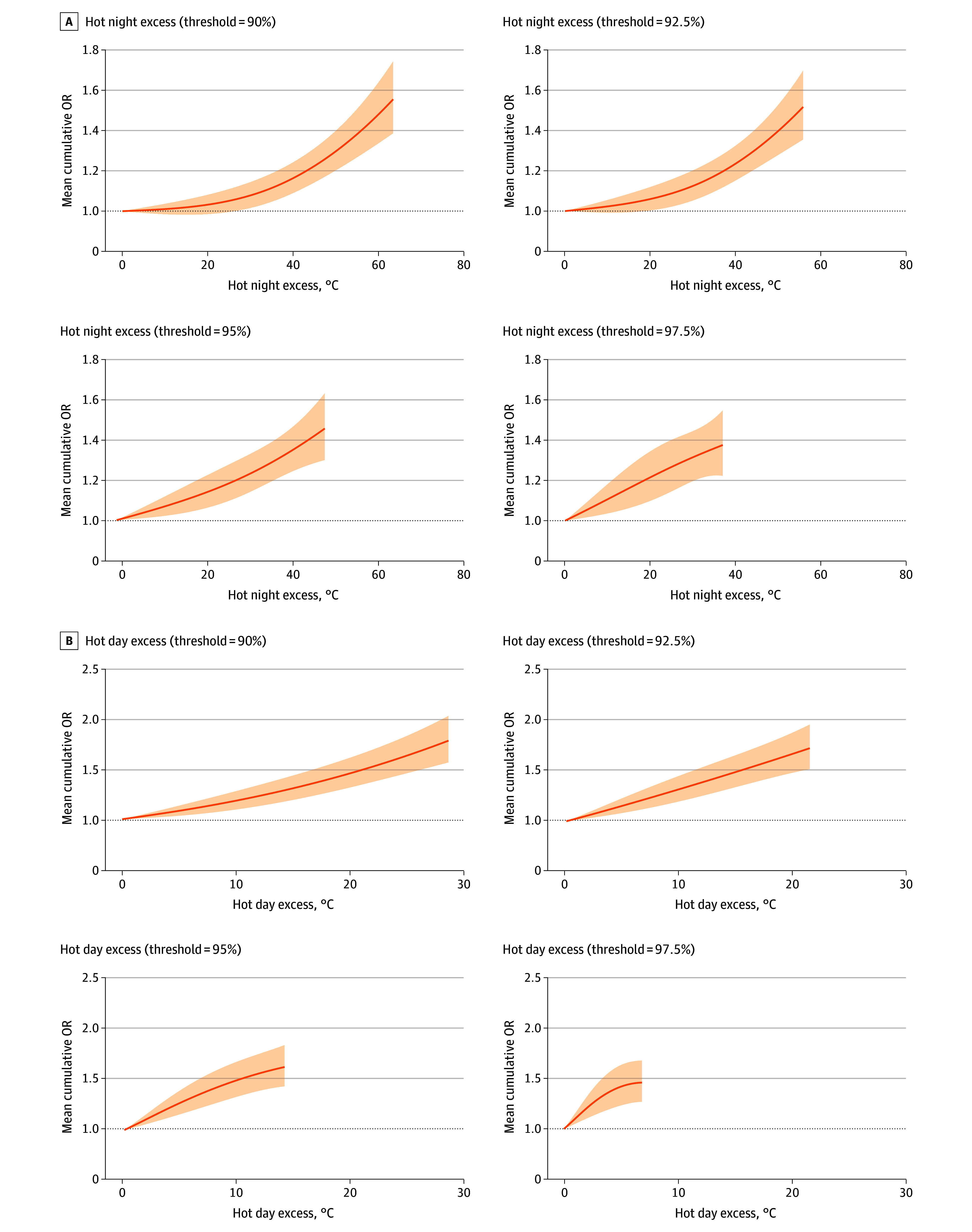
Exposure-Response Curves for the Association Between Hot Excess and Dementia-Related Deaths These metrics were calculated based on the amount by which observed nighttime or daytime temperatures exceeded the locally defined threshold. Solid lines indicate the mean cumulative odds ratios (ORs) of dementia-related deaths with specific hot excess over a lag of 0 to 13 days. Shaded areas indicate the 95% CIs.

Figure 1 illustrates the exposure-response curves for different definitions of HNE and HDE in association with dementia-related deaths. These curves share a similar linear shape, indicating a higher risk of dementia-related deaths associated with a larger magnitude of heat without any discernible thresholds. Accordingly, the reference values for subsequent analyses were set at the 1st percentile of HNE and HDE that approximates the lowest risk.

**Figure 2.**
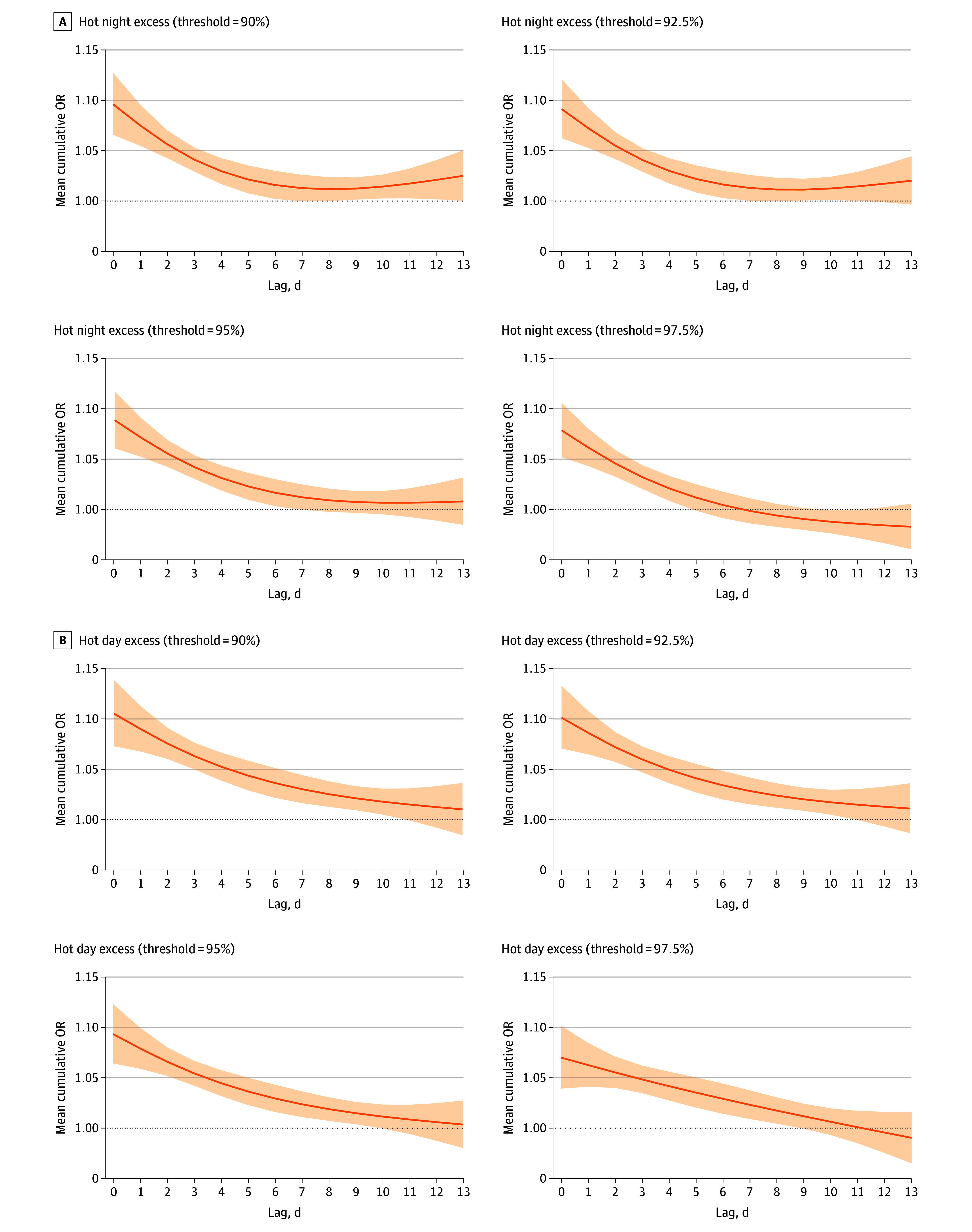
Lag-Response Curves for the Association Between Extreme Hot Excess and Dementia-Related Deaths These metrics were calculated based on the amount by which observed nighttime or daytime temperatures exceeded the locally defined threshold. Solid lines indicate the mean cumulative odds ratios (ORs) of dementia-related deaths with extreme hot excess over a lag of 0 to 13 days. Shaded areas indicate the 95% CIs.

Figure 2 displays the lag patterns for the risk of dementia-related deaths associated with HNE and HDE of various definitions. The associations of hot nights with risk of dementia-related deaths was most pronounced on the same day, gradually decreased, and became statistically nonsignificant after a lag of 6 days. In contrast, the association of hot days with risk of dementia-related deaths was highest on the same day, followed by a gradual decrease, and became statistically insignificant after a lag of 10 days.

Table 1 presents the ORs and AFs of dementia-related deaths in association with extreme HNE and HDE under different threshold definitions. Extreme HDE exhibited a stronger relative risk compared with extreme HNE. Hot day excess had a larger burden for definitions at 90% and 92.5%, while HNE had a larger burden as the definitions became more stringent. Relative to the reference hot excess, the OR for dementia-related deaths associated with extreme HNE was 1.56 (95% CI, 1.39-1.74) for a threshold definition at 90%, 1.52 (95% CI, 1.36-1.70) at 92.5%, 1.46 (95% CI, 1.30-1.63) at 95%, and 1.38 (95% CI, 1.22-1.55) at 97.5%. The AF for HNE was 3.23% (95% eCI, 3.17%-3.27%) for a threshold definition at 90%, 2.79% (95% eCI, 2.74%-2.82%) at 92.5%, 2.46% (95% eCI, 2.42%-2.48%) at 95%, and 1.45% (95% eCI, 1.43%-1.47%) at 97.5%. Regarding extreme HDE, the OR was 1.79 (95% CI, 1.57-2.04) for a threshold definition at 90%, 1.72 (95% CI, 1.52-1.96) at 92.5%, 1.62 (95% CI, 1.43-1.83) at 95%, and 1.46 (95% CI, 1.27-1.68) at 97.5%, with AFs of 3.43% (95% eCI, 3.37%-3.46%) for a threshold definition at 90%, 3.02% (95% eCI, 2.98%-3.04%) at 92.5%, 2.26% (95% eCI, 2.23%-2.28%) at 95%, and 1.10% (95% eCI, 1.08%-1.11%) at 97.5%.

**Table 1.** Dementia-Related Deaths Associated With Extreme Hot Excess^a^

Threshold, %	Hot night excess		Hot day excess	
	Odds ratio (95% CI)	Attributable fraction, % (95% CI)	Odds ratio (95% CI)	Attributable fraction, % (95% CI)
90	1.56 (1.39-1.74)	3.23 (3.17-3.27)	1.79 (1.57-2.04)	3.43 (3.37-3.46)
92.5	1.52 (1.36-1.70)	2.79 (2.74-2.82)	1.72 (1.52-1.96)	3.02 (2.98-3.04)
95	1.46 (1.30-1.63)	2.46 (2.42-2.48)	1.62 (1.43-1.83)	2.26 (2.23-2.28)
97.5	1.38 (1.22-1.55)	1.45 (1.43-1.47)	1.46 (1.27-1.68)	1.10 (1.08-1.11)

^a^ The associations are presented as cumulative odds ratios (mean and 95% CI) of dementia-related deaths comparing the extreme hot excess with the reference values over a lag of 0 to 13 days.

Regarding dementia subtype analysis, unspecified dementia showed a higher risk with nighttime high temperatures, while Alzheimer disease exhibited a stronger association with daytime high temperatures (Table 2). In Table 2, we present the results of stratified analyses by age, sex, educational level, and region. Our findings indicate a significant increase in the risk of dementia-related deaths for both daytime and nighttime heat among female decedents, individuals aged 75 years or older, and those with lower educational levels. Furthermore, regional analysis revealed that the risk associated with nighttime heat was more prominent in the south, whereas the risk associated with daytime heat was heightened in the north.

**Table 2.** Associations Between Extreme Hot Excess and Dementia-Related Deaths in Multiple Subgroups^a^

Subgroup	Odds ratio (95% CI)	
	Hot night excess	Hot day excess
Overall	1.46 (1.30-1.63)	1.62 (1.43-1.83)
Dementia subtype		
Alzheimer disease	1.35 (1.06-1.72)	1.76 (1.36-2.27)
Vascular dementia	1.39 (1.01-1.90)	1.31 (0.92-1.88)
Unspecified dementia	1.50 (1.30-1.74)	1.60 (1.37-1.87)
Age, y		
<65	1.05 (0.67-1.66)	1.09 (0.67-1.77)
≥65 to ≤74	1.18 (0.86-1.63)	1.61 (1.13-2.28)
≥75	1.53 (1.34-1.73)	1.66 (1.44-1.91)
Sex		
Male	1.39 (1.18-1.65)	1.45 (1.20-1.74)
Female	1.54 (1.32-1.80)	1.77 (1.49-2.09)
Educational level		
Middle school and below	1.47 (1.31-1.66)	1.65 (1.45-1.87)
High school and above	0.99 (0.63-1.55)	1.02 (0.61-1.72)
Region		
South	1.65 (1.42-1.93)	1.55 (1.32-1.81)
North	1.11 (0.91-1.34)	1.60 (1.29-1.99)

^a^ The associations are presented as cumulative odds ratios (mean and 95% CI) of dementia-related deaths comparing the extreme hot excess with the reference values over a lag of 0 to 13 days.

Sensitivity analyses showed no significant alterations in the main estimates after controlling for the concomitant exposure to air pollution. When HNE and HDE were simultaneously included in the model, the risk associated with HNE decreased more prominently than the risk associated with HDE, but both were statistically significant (Table 3).

**Table 3.** Associations Between Extreme Hot Excess and Dementia-Related Deaths in Multiple Sensitivity Analyses^a^

Threshold, %	Odds ratio (95% CI)			
	Hot night excess		Hot day excess	
	With adjustment of PM_2.5_ and O_3_^b^	With adjustment of hot day excess^c^	With adjustment of PM_2.5_ and O_3_^b^	With adjustment of hot night excess^d^
90	1.56 (1.38-1.76)	1.28 (1.12-1.45)	1.72 (1.49-1.99)	1.64 (1.43-1.88)
92.5	1.54 (1.36-1.74)	1.27 (1.12-1.44)	1.68 (1.46-1.93)	1.59 (1.39-1.82)
95	1.49 (1.31-1.68)	1.24 (1.09-1.40)	1.59 (1.39-1.83)	1.48 (1.30-1.69)
97.5	1.41 (1.24-1.60)	1.22 (1.07-1.38)	1.45 (1.24-1.69)	1.34 (1.16-1.56)

Abbreviations: O_3_, ozone; PM_2.5_, particulate matter with aerodynamic diameter of 2.5 μm or smaller.

^a^ The associations are presented as cumulative odds ratios (mean and 95% CI) of dementia-related deaths comparing the extreme hot excess with the reference values over a lag of 0 to 13 days.

^b^ The model further controls for fine PM_2.5_ and O_3_ with a lag of 0 to 2 days.

^c^ The model further controls for hot day excess at the same lag.

^d^ The model further controls for hot night excess at the same lag.

## Discussion

This nationwide case-crossover study found that both nighttime and daytime heat are associated with increased risk of dementia-related deaths. The association of hot nights with risk of dementia-related deaths persisted for 6 days, while hot days exhibited a prolonged association with risk of dementia-related deaths over a 10-day period. Subgroup analyses reveal heightened vulnerability in association with specific demographic characteristics, including women, individuals aged 75 years or older, and those with lower educational levels. Furthermore, regional disparities are evident, with greater sensitivity to nighttime heat in the south and larger vulnerability to daytime heat in the north. Nighttime heat with stricter thresholds was associated with a more substantial burden of dementia-related deaths. These findings underscore the importance of tailored public health interventions at different times of the day, which is particularly critical for aging populations to effectively mitigate risk associated with extreme heat.

Previous studies have reported associations between ambient high temperatures and dementia-related outcomes.^10,11,12,13,14^ Concurrently, high nighttime temperature has been identified as a significant and independent health risk factor, particularly for older adults.^22,23,24,25^ However, our study distinguishes itself as the first, to our knowledge, to systematically differentiate the associations of nighttime and daytime heat with dementia-related deaths. Our findings reveal that both nighttime and daytime heat have an immediate and significant association with dementia-related deaths. Furthermore, our study identifies distinct associations of nighttime and daytime heat with dementia-related deaths, highlighting a greater burden associated with extreme nighttime heat. This finding underscores the importance of recognizing these 2 factors as distinct risk contributors. Furthermore, we found varied risks of daytime and nighttime heat with different subtypes of dementia-related deaths, implying that different types of dementia may have unique responses to the timing and intensity of heat exposure.

The differential associations of hot nights and hot days with dementia-related deaths stem from distinct physiological mechanisms influenced by temporal variations in temperature exposure and individual behavior. Specifically, discomfort-induced sleep disruption during hot nights may amplify cognitive decline among individuals with dementia given the pivotal role of sleep in cognition.^26,32^ In addition, nighttime heat poses an increased risk of dehydration, especially among older adults, exacerbating cognitive decline.^33^ Moreover, elevated nighttime temperatures can be associated with heightened agitation and anxiety, compounding cognitive and emotional challenges.^34,35^ Conversely, during the day, individuals are more likely to engage in outdoor activities, exposing themselves to direct sunlight and elevated temperatures. This heightened physical activity can result in increased heat production within the body, leading to mechanisms such as enhanced sweating and blood flow to dissipate heat. However, in the case of individuals with dementia, impaired cognitive function may impede their ability to recognize the need for hydration and rest, potentially exacerbating physiological stress caused by daytime heat.^36^ Individuals with dementia may also experience a diminished ability to perceive changes in temperature,^37^ leading to reduced sensitivity to heat cues and resulting in delayed or inadequate responses to environmental heat stress. Consequently, they may not take appropriate measures to cool themselves down, such as seeking shade or increasing fluid intake, putting them at higher risk during both daytime and nighttime heat events.

Our stratified analyses reveal varied vulnerabilities to the association of extreme heat with dementia-related deaths across different population subgroups. Individuals aged 75 years or older are notably susceptible due to age-related declines in temperature regulation, preexisting chronic conditions, and reduced mobility.^38^ In addition, women show heightened sensitivity, likely influenced by factors such as menopause and sex-specific differences in thermoregulation. Postmenopausal women, experiencing hormonal fluctuations, may face disrupted thermoregulation, potentially increasing their susceptibility to heat.^39,40^ Moreover, inherent differences in body composition and thermoregulatory mechanisms between sexes may be associated with the elevated risk among women.^41^ Individuals with lower educational levels also face elevated vulnerability, which may be associated with socioeconomic factors such as limited resources, knowledge gaps, and the urban heat island effect (where urban areas retain and produce heat while having less plant life to dissipate it).^42^ Regional disparities in vulnerability may stem from a combination of climate, infrastructure, and cultural factors.^43^ Specifically, the south shows greater sensitivity to nighttime heat, which may be due to its warmer climate and higher humidity levels. These findings underscore the importance of tailored interventions for specific subpopulations to mitigate dementia-related burden during extreme heat events.

Our study carries notable implications for public health, particularly in response to the increasing frequency of heat events associated with climate change. We emphasize the urgent need for action to protect vulnerable populations, such as elderly individuals with dementia. Effective measures are crucial, including improved access to cooling facilities, heightened public awareness of heat-related risks, and strengthened health care infrastructure to manage heat-related health issues. Health care systems must be prepared for heightened demand during extreme heat events, especially among patients with dementia. Understanding the lag patterns of heat effects can optimize resource allocation and ensure timely care delivery. Tailored interventions addressing the specific needs of vulnerable groups identified in our study are essential to provide adequate support and attention to those at higher risk. Policy makers should consider temporal variations in heat vulnerability when developing climate adaptation strategies. Recognizing the unique challenges posed by extreme heat at different times, policy makers should tailor approaches accordingly. Our findings underscore the imperative for time- and population-specific public health measures. These measures should be integrated into broader climate change mitigation and adaptation policies to safeguard the health of the most susceptible populations now and in the future.

### Strengths and Limitations

Our study has some strengths, including its nationwide scope, use of a case-crossover design to minimize potential confounders, and the comprehensive dataset, providing a more thorough and accurate understanding of the temperature-dementia association. There are also several limitations in our study. First, although we covered all mainland China regions, caution is warranted when extrapolating findings to countries with different socioeconomic characteristics. Second, we relied on simulated ambient temperature data rather than individual measurements, which may introduce exposure errors. Third, despite rigorous data verification, diagnostic errors are inevitable in a nationwide registry due to the complexity of dementia diagnoses. Fourth, the absence of data on individual use of medication and air conditioning restricted our exploration of their modifying associations with the risk of heat-related deaths among individuals with dementia.

## Conclusions

This nationwide case-crossover study reveals novel evidence that both nighttime and daytime heat are associated with increased risk of dementia-related deaths, highlighting a greater burden associated with nighttime heat. These findings emphasize the necessity for tailored public health interventions at various times of the day, which is especially crucial among an aging population to effectively alleviate risk associated with heat exposure.

## Notes

Supplement 1. **eMethods 1.** The Definitions of “Daytime” and “Nighttime” **eMethods 2.** Introduction of Individual-Level, Time-Stratified Case-Crossover Design **eMethods 3.** The Calculation Procedure of Backward Attributable Fraction (AF) and Number (AN) **eTable 1.** The Summary Statistics of Dementia-Related Deaths in China Mainland During the Study Period **eFigure.** Maps of the Different Defined Hot Night and Hot Day Conditions in Mainland China During the Study Period **eTable 2.** Overview of the Hot Night Condition in all Counties from China Mainland During the Study Period **eTable 3.** Overview of the Hot Day Condition in all Counties from China Mainland During the Study Period
eReferences

Supplement 2.
Data Sharing Statement
