# Supplementary material for: Heat Exposure and Dementia-Related Mortality in China
Source: JAMA Netw Open. 2024 Jun 28;7(6):e2419250. doi: 10.1001/jamanetworkopen.2024.19250 (PMC11214125; doi:10.1001/jamanetworkopen.2024.19250)
Supplement: Supplement 1. — eMethods 1. The Definitions of “Daytime” and “Nighttime” eMethods 2. Introduction of Individual-Level, Time-Stratified Case-Crossover Design eMethods 3. The Calculation Procedure of Backward Attributable Fraction (AF) and Number (AN) eTable 1. The Summary Statistics of Dementia-Related Deaths in China Mainland During the Study Period eFigure. Maps of the Different Defined Hot Night and Hot Day Conditions in Mainland China During the Study Period eTable 2. Overview of the Hot Night Condition in all Counties from China Mainland During the Study Period eTable 3. Overview of the Hot Day Condition in all Counties from China Mainland During the Study Period eReferences [file jamanetwopen-e2419250-s001.pdf]

Supplementary Online Content

Gao Y, Lin L, Yin P, Kan H, Chen R, Zhou M. Heat and dementia-related mortality in China. *JAMA Netw Open.* 2024;7(6):e2419250. doi:10.1001/jamanetworkopen.2024.19250

eMethods 1. The Definitions of "Daytime" and "Nighttime"
eMethods 2. Introduction of Individual-Level, Time-Stratified Case-Crossover Design
eMethods 3. The Calculation Procedure of Backward Attributable Fraction (AF) and Number (AN)
eTable 1. The Summary Statistics of Dementia-Related Deaths in China Mainland During the Study Period
eFigure. Maps of the Different Defined Hot Night and Hot Day Conditions in Mainland China During the Study Period
eTable 2. Overview of the Hot Night Condition in all Counties from China Mainland During the Study Period
eTable 3. Overview of the Hot Day Condition in all Counties from China Mainland During the Study Period
eReferences.

This supplementary material has been provided by the authors to give readers additional information about their work.

© 2024 Gao Y et al. *JAMA Network Open.*

eMethods 1. The Definitions of "Daytime" and "Nighttime"

In our study, we established the definitions of "daytime" and "nighttime" for each county based on local sunrise and sunset time, which vary daily and geographically. Geographically specific sunrise and sunset time were calculated for the central point of each county using the "suncalc" package (version 0.5.1) in R software. This package calculates solar positions based on latitude, longitude, and date. Specifically, daytime was defined as the period from one hour after sunrise to one hour before sunset, encompassing the hours of daylight. Conversely, nighttime was defined as the period from one hour after sunset to one hour before the following sunrise, covering the hours of darkness. These definitions allowed for consistent characterization of daytime and nighttime across different locations and dates within the study period.

© 2024 Gao Y et al. *JAMA Network Open.*

eMethods 2. Introduction of Individual-Level, Time-Stratified Case-Crossover Design

The individual-level, time-stratified case-crossover is a variant of the case-control study and has been widely used in the field of environmental epidemiology.[1-3] The design effectively controls for individual-level risk factors, such as demographic, socioeconomic, and behavioral factors, by self-matching. It also automatically removes temporal trends by selecting control groups within the same month. In our analysis, the case day was defined as the day of death. We then matched each case day with 3 or 4 control days in the same year, month, and day of the week to account for both long- and short-term variations and seasonality. For instance, if the date of death was on Tuesday, January 15, 2019, Tuesday, January 15, 2019, would be the case day, and all other Tuesdays in January 2019 (i.e., June 1, 8, 22, and 29) would serve as control days.

© 2024 Gao Y et al. *JAMA Network Open.*

eMethods 3. The Calculation Procedure of Backward Attributable Fraction (AF) and Number (AN)

To assess the burden of dementia-related deaths associated with various definitions of HNE and HDE, we adopted a backward-looking approach as previously proposed.[4,5] This approach offers precise estimations of the disease burden by accounting for complex lag patterns in temperature-related risks. The backward attributable fraction (AF) and attributable number (AN) were calculated using the following formulas[4,6]:

$$AF_{x,t} = 1 - \exp\left(-\sum_{\ell=\ell_0}^{L} \beta_{x_{(t-\ell)},\ell}\right) \quad (1)$$

$$AN_{x,t} = AF_{x,t} \times n_t \quad (2)$$

Here, n_t represents the number of death cases at time t. $AN_{x,t}$ and $AF_{x,t}$ indicate the number of death cases and the related fractions at time t attributable to a series of exposures $x_{(t-\ell 0)}, \ldots, x_{(t-L)}$ experienced over the lag period $\ell = \ell_0, \ldots, L$, compared to the referent exposure. ℓ_0 and L corresponding to minimum and maximum lags, respectively.

Initially, we computed the AN for each county based on the exposure-response associations. Subsequently, we derived the national AF by summing the ANs for all counties and dividing by the total number of dementia-related deaths across the entire country. To estimate empirical confidence intervals (eCIs) for AF, we conducted Monte Carlo simulations, assuming a multivariate normal distribution of point estimates and utilizing the covariance matrix derived from the regression model. The 2.5th and 97.5th percentiles of the resulting distributions from simulating 1,000 random samples were interpreted as the 95% eCIs.[4,6]

© 2024 Gao Y et al. *JAMA Network Open.*

eTable 1. The Summary Statistics of Dementia-Related Deaths in China Mainland During the Study Period

Subgroup	Sample size (N)	Proportion (%)
Overall	132,573	100.0
Dementia subtype		
Alzheimer disease	31,804	24.0
Vascular dementia	16,873	12.7
Unspecified dementia	83,896	63.3
Age		
<65	8,242	6.2
≥65 to ≤74	16,189	12.2
≥75	108,142	81.6
Sex		
Male	59,487	44.9
Female	73,086	55.1
Education		
Middle school and below	124,098	93.6
High school and above	7,901	6.0
Region		
South	83,632	63.1
North	48,941	36.9

Note: The percentages represent the proportion of individuals with available data for each category.

© 2024 Gao Y et al. *JAMA Network Open.*

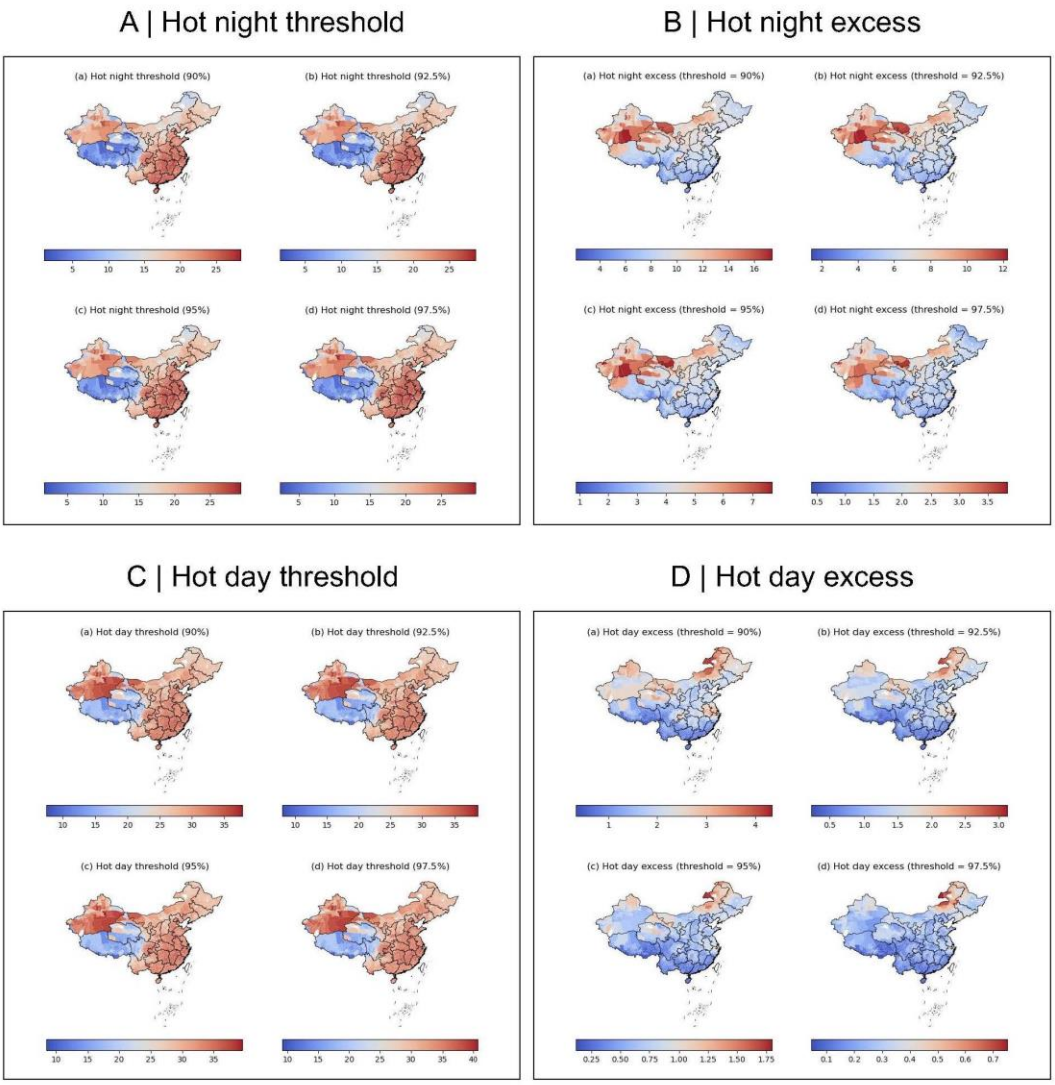

eFigure. Maps of the Different Defined Hot Night and Hot Day Conditions in Mainland China During the Study Period

Note: The hot night threshold is calculated as a specific percentage of the daily minimum temperature for the study period. The hot day threshold is calculated as a specific percentage of the daily maximum temperature for the study period. All values were calculated by hourly temperature series from ERA5 dataset.

© 2024 Gao Y et al. *JAMA Network Open.*

eTable 2. Overview of the Hot Night Condition in all Counties from China Mainland During the Study Period

Threshold	Hot night threshold (Median [IQR]) (unit: ℃)	Hot night excess (HNE) (Median [IQR]) (unit: ℃)
Threshold = 90%	23.2 (18.8, 25.0)	8.8 (7.1, 9.9)
Threshold = 92.5%	23.8 (19.4, 25.5)	6.0 (5.0, 7.0)
Threshold = 95%	24.5 (20.1, 26.2)	3.7 (3.1, 4.3)
Threshold = 97.5%	25.3 (21.1, 27.1)	1.7 (1.4, 2.0)

Note: The hot night threshold is calculated as a specific percentage of the daily minimum temperature for the study period. All values were calculated by hourly temperature series from ERA5 dataset, IQR, interquartile range.

© 2024 Gao Y et al. *JAMA Network Open.*

eTable 3. Overview of the Hot Day Condition in all Counties from China Mainland During the Study Period

Threshold	Hot day threshold (Median [IQR]) (unit: ℃)	Hot day excess (HDE) (Median [IQR]) (unit: ℃)
Threshold = 90%	31.8 (28.5, 33.2)	1.9 (1.4, 2.3)
Threshold = 92.5%	32.5 (29.1, 33.9)	1.2 (0.9, 1.4)
Threshold = 95%	33.3 (29.9, 34.7)	0.6 (0.5, 0.8)
Threshold = 97.5%	34.2 (31.1, 35.8)	0.2 (0.2, 0.3)

Note: The hot day threshold is calculated as a specific percentage of the daily minimum temperature for the study period. All values were calculated by hourly temperature series from ERA5 dataset, IQR, interquartile range.

eReferences.

1. Shahn Z, Hernán MA, Robins JM. A formal causal interpretation of the case-crossover design. *Biometrics* 2023; **79**(2): 1330-43.
2. Tobias A, Kim Y, Madaniyazi L. Time-stratified case-crossover studies for aggregated data in environmental epidemiology: a tutorial. *International Journal of Epidemiology* 2024; **53**(2): dyae020.
3. Chen J, Gao Y, Jiang Y, et al. Low ambient temperature and temperature drop between neighbouring days and acute aortic dissection: a case-crossover study. *Eur Heart J* 2022; **43**(3): 228-35.
4. Gasparrini A, Leone M. Attributable risk from distributed lag models. *BMC medical research methodology* 2014; **14**(1): 1-8.
5. Zhou Y, Gao Y, Yin P, et al. Assessing the burden of suicide death associated with nonoptimum temperature in a changing climate. *JAMA psychiatry* 2023; **80**(5): 488-97.
6. Faustini A, Davoli M. Attributable risk to assess the health impact of air pollution: advances, controversies, state of the art and future needs. *International Journal of Environmental Research and Public Health* 2020; **17**(12): 4512.

© 2024 Gao Y et al. *JAMA Network Open.*
