# Supplementary material for: Heat Exposure and Dementia-Related Mortality in China
Source: JAMA Netw Open. 2024 Jun 28;7(6):e2419250. doi: 10.1001/jamanetworkopen.2024.19250 (PMC11214125; doi:10.1001/jamanetworkopen.2024.19250)
Supplement: Supplement 2. — Data Sharing Statement [file jamanetwopen-e2419250-s002.pdf]

Data Sharing Statement

Gao. Heat Exposure and Dementia-Related Mortality in China. *JAMA Netw Open*. Published June 28, 2024. doi:10.1001/jamanetworkopen.2024.19250

Data
Data available: No

Additional Information
Explanation for why data not available: Code and data sources are available from the corresponding author upon request.
